# Rural youth’s exposure to firearm violence and their attitudes regarding firearm safety measures

**DOI:** 10.1186/s40621-021-00317-x

**Published:** 2021-09-13

**Authors:** Charles A. Jennissen, Ryan P. King, Kristel M. Wetjen, Gerene M. Denning, Cole C. Wymore, Nicholas R. Stange, Pamela J. Hoogerwerf, Junlin Liao, Kelly E. Wood

**Affiliations:** 1 Department of Emergency Medicine, Roy J. and Lucille A. Carver College of Medicine, University of Iowa, Iowa City, USA; 2 Stead Family Department of Pediatrics, Roy J. and Lucille A. Carver College of Medicine, University of Iowa, Iowa City, USA; 3 Division of Pediatric Surgery, Roy J. and Lucille A. Carver College of Medicine, University of Iowa, Iowa, USA; 4 Saint Louis University School of Medicine, Saint Louis University, St. Louis, MO USA; 5 Injury Prevention and Community Outreach, University of Iowa Stead Family Children’s Hospital, University of Iowa, Iowa City, USA; 6 Department of Surgery, Roy J. and Lucille A. Carver College of Medicine, University of Iowa, Iowa, USA

**Keywords:** Firearms, Rifles, Shotguns, Handguns, Youth, Rural, Adolescents, Gun violence prevention, Gun control, Attitudes

## Abstract

**Background:**

In the wake of an epidemic in firearm-related deaths and injuries, youth have become leading voices of concern. This study’s objective was to investigate rural youth’s personal experiences with firearm-related violence, and their attitudes towards firearms and gun violence prevention strategies.

**Methods:**

Attendees of the 2019 Iowa FFA Leadership Conference were surveyed about personal experiences with firearm-associated deaths and injuries, and their attitudes regarding firearm-related issues. Descriptive (frequencies), bivariate (chi square, Fisher’s exact test) and multivariable logistic regression analyses were performed utilizing Stata 15.1 (StataCorp, College Station, Texas).

**Results:**

Responses from 1382 FFA members 13–18 years of age were analyzed. About 5% had personally seen someone threatened with a firearm. Over one-third (36%) stated they knew someone who had been killed or injured by gunfire. Of these, over two-thirds knew of someone who had died or was injured unintentionally and 30% knew of someone killed or injured intentionally (e.g. suicide). Nearly all agreed or strongly agreed that the right to use firearms for hunting and shooting sports should be legal (94%), that a firearm safety course should be required to get a hunting license (89%), and that there should be a required background check before purchasing a firearm (89%). Over three-fifths (61%) agreed or strongly agreed that there should be laws requiring safe storage of firearms in homes. Although still high, lesser support for firearm safety policies was seen among males, older youth, participants living on farms or in the country, and youth who hunted, had firearms in their homes, and/or were in homes with unsafe firearm storage.

**Conclusions:**

The majority of youth in this study supported firearm safety measures including required training, background checks, and safe firearm storage in homes. These findings are consistent with the national youth-led call for firearm safety. Additionally, over one-third of respondents personally knew someone who was killed or injured by a firearm and 5% had seen someone or been personally threatened with firearm violence. Our study did not investigate the effects of firearm violence on participants’ mental health and wellbeing, but future studies addressing this question seem highly justified.

## Background

From 1999 to 2017, over 600,000 people died in the United States (U.S.) from firearm-related violence (Goldstick et al., 2019), and the age-adjusted mortality rate from firearms in 2018 was 11.9 per 100,000 people (Goldstick & Cunningham, 2020). As compared to other developed countries, the U.S. has a firearm-related homicide rate, suicide rate, and unintentional death rate around 25 times, 8 times, and 6 times higher, respectively (Grinshteyn & Hemenway, 2016). In addition, the annual number of survivable injuries is twice that of deaths (Fowler et al., 2015).

In the U.S., youth mortality due to firearms is the highest in the world (Centers for Disease Control and Prevention, 1997), and firearms are one of the top three causes of pediatric deaths (Dowd & Sege, 2012). In fact, 91% of all childhood firearm-related deaths (0–14 years) in high-income countries occur in the U.S. (Grinshteyn & Hemenway, 2016). Firearms are also the leading cause of death among youth 14–18 years old, responsible for 25% more fatalities than motor vehicle crashes (Goldstick & Cunningham, 2020).

With respect to rural versus urban youth, when U.S. firearm-related mortality recently declined 2.7% overall among youth 0–19 years of age, it increased 11.4% in rural areas (Goldstick & Cunningham, 2020). Additionally, rural children 5–14 years were more than twice as likely as urban children to be admitted for unintentional firearm injuries (Herrin et al., 2018). A national study further showed that the firearm suicide rate was significantly higher and the firearm homicide rate was significantly lower in rural as compared to urban counties (Branas et al., 2004). Several other studies also found higher rates of suicide and unintentional firearm-related deaths in rural as compared to urban areas (Nance et al., 2010; Carr et al., 2012; Fontanella et al., 2015; Dodge et al., 1994; Guetschow et al., 2018).

In the wake of ongoing mass shootings in U.S. schools and the rise in adolescent suicides, youth have become leaders in calling for an increased public focus on gun violence prevention and policy reform. Although the opinions of adults regarding firearms and gun laws have been tracked by national polls for decades (Poll, 2019), few media polls or research studies have addressed the viewpoint of youth. The objectives of this study were to ascertain rural youth’s personal experience with firearm-related violence, and their attitudes toward firearms and gun violence prevention strategies.

## Methods

### Study population

A survey was administered to a convenience sample of FFA members attending the 2019 Iowa FFA Leadership Conference. FFA (formerly Future Farmers of America) is an intra-curricular high school organization for students interested in agriculture and leadership. As of 2019, there were 15,644 members in 246 Iowa FFA chapters (Iowa FFA Association and the Iowa Council on Agricultural Education, 2020). Conference attendees were recruited to complete the survey at the University of Iowa Stead Family Children’s Hospital safety booth. Final analysis was performed using surveys completed by participants 13–18 years of age. The authors’ Institutional Review Board deemed this study exempt as the research analysis was done on an existing dataset that had been collected anonymously.

### Survey

The survey was developed through a collaborative and iterative process among members of the University of Iowa Stead Family Children’s Hospital’s Injury Prevention Program and other individuals interested in firearm injury prevention at the University of Iowa Hospital and Clinics. The written survey was administered to 20 youth and young adults 11–22 years of age for validation. After completion, participants were asked to explain and to clarify their responses if a question was not easily understood. The written and verbal responses provided were compared for consistency. Final survey design reflected these validation results.

Demographic variables included age, sex, residence type (e.g. on a farm), and race/ethnicity. The five individuals who noted their sex as “other” were excluded from comparative analysis. Due to limited numbers of several groups, race/ethnicity was dichotomized to Caucasian and non-Caucasian. This resulted in significant heterogeneity within the non-Caucasian group but allowed use of the variable in comparative analyses. Responses to the presence of rifles/shotguns and handguns in the home were “Yes” or “Not that I know of”.

Questions related to a participant’s exposure to firearm-related violence included “Have you personally seen anyone being threatened with a firearm (without anyone getting injured or killed)?” and if so, who was that person (family, friends, myself, others). Respondents had the option of checking all that applied. Participants were also asked, “Do you personally know of anyone who was injured or killed by gunfire?” and if so, was that injury/death “by accident” or “on purpose”, and who was the person injured or killed.

Respondents were then asked to indicate how much they agreed or disagreed with each of the following statements: 1) The right to use firearms for hunting and shooting sports should be kept legal; 2) A firearm safety course should be required to get a hunting license; 3) There should be a background check required by law before someone can buy a firearm; and 4) There should be laws that require safe storage (locked and unloaded) of firearms in homes. Answers included strongly agree, agree, neither agree nor disagree, disagree, and strongly disagree.

Other questions used for comparative analysis included those on the storage of firearms in their home, experience with using firearms (e.g. hunting), and whether they had taken a formal/certified hunter or firearm safety training course. Choices for the frequency of lifetime firearm use were never, 1–5 times, 6–20 times, and > 20 times. For questions on firearm storage, “safe storage” was defined as every firearm in the home always stored locked and unloaded. All other conditions were defined as “unsafe storage”, and respondents were given the option to choose unsure for storage-related questions.

### Data analysis

Responses on paper surveys were entered into Qualtrics™. Data were then exported as an Excel spreadsheet and imported into Stata 15.1 (StataCorp, College Station, Texas) for descriptive (frequencies), bivariate (chi-square, Fisher’s exact test) and multivariable logistic regression analyses. All *p*-values were two-tailed and a value < 0.05 was considered statistically significant. Missing data were not included in analyses.

## Results

### Demographics and firearms in the home

A total of 1382 FFA members 13–18 years old completed the survey. See Table 1. The population was roughly equal by sex and about one-third (35%) were 13–15 years old. Over half lived on a farm, almost one-fifth resided in the country but not on a farm, and 29% lived in a town. The vast majority self-identified as Caucasian (96%). Most survey youth had firearms in their home including rifles/shotguns (84%) and handguns (58%).

**Table 1:** Demographics and firearm access among respondents of the Iowa FFA firearm survey

	*n* (Col %)^a^
Group N	1382
Sex	
Male	697 (50%)
Female	680 (49%)
Other	5 (< 1%)
Age	
13 years	29 (2%)
14 years	120 (9%)
15 years	330 (24%)
16 years	363 (26%)
17 years	321 (23%)
18 years	219 (16%)
Residence	
Farm	727 (53%)
Country/Not Farm	250 (18%)
Town	400 (29%)
Race	
Caucasian	1320 (96%)
Non-Caucasian	61 (4%)
Rifle/Shotgun in Home	
Yes	1159 (84%)
Not That I Know Of	223 (16%)
Handgun in Home	
Yes	802 (58%)
Not That I Know Of	580 (42%)

^a^ The sum of n may not equal the total Group N due to missing values

### Witnessed someone threatened with a firearm

Five percent of youth participants reported personally witnessing someone being threatened with a firearm. See Table 2. Of these, 38% (24/64) had seen a friend and 36% (23/64) had seen a family member threatened. Over one-third (23/64, 36%) had seen someone other than family and/or friends threatened, and 11% (7/64) had personally been the target of a threat with a firearm.

**Table 2:** Bivariate analyses of personal experience with firearm violence among respondents of the Iowa FFA survey as a function of demographics and presence of firearms in the home

	Personally Seen Someone Threatened with a Firearm			Personally Know of Someone Killed or Injured by a Firearm		
	Yes *n* (Row %)^a^	No *n* (Row %)^a^	*p* value	Yes *n* (Row %)^a^	No *n* (Row %)^a^	*p* value
Group N	64 (5%)	1317 (95%)		493 (36%)	887 (64%)	
Sex			0.82			0.006
Male	31 (4%)	665 (96%)		224 (32%)	471 (68%)	
Female	32 (5%)	648 (95%)		267 (39%)	413 (61%)	
Age			0.82			0.28
16–18 years	41 (5%)	862 (95%)		331 (37%)	570 (63%)	
13–15 years	23 (5%)	455 (95%)		162 (34%)	317 (66%)	
Residence			0.18			0.065
Farm	38 (5%)	689 (95%)		239 (33%)	487 (67%)	
Country/Not Farm	14 (6%)	236 (94%)		100 (40%)	150 (60%)	
Town	12 (3%)	387 (97%)		152 (38%)	247 (62%)	
Race			0.059			0.25
Caucasian	58 (4%)	1261 (96%)		467 (35%)	851 (65%)	
Non-Caucasian	6 (10%)	55 (90%)		26 (43%)	35 (57%)	
Rifle/Shotgun in Home			0.82			0.025
Yes	53 (5%)	1105 (95%)		428 (37%)	729 (63%)	
No	11 (5%)	212 (95%)		65 (29%)	158 (71%)	
Handgun in Home			0.005			0.020
Yes	48 (6%)	754 (94%)		307 (38%)	495 (62%)	
No	16 (3%)	563 (97%)		186 (32%)	392 (68%)	

^a^ The sum of n for a variable may not equal the total Group *N* due to missing values

In bivariate analysis, those with a handgun in the home had higher percentages having seen someone personally threatened with a firearm as compared to those with no handguns in the home (*p* = 0.005). This relationship was not seen with rifles/shotguns in the home. See Table 2. Logistic regression analysis indicated that non-Caucasian races when grouped were 2.6 times more likely than Caucasians and that respondents with handguns in the home were 3 times more likely than those without to have witnessed the threat of firearm violence. See Table 3.

**Table 3:** Logistic regression analyses of personal experience with firearm violence among respondents of the Iowa FFA firearm survey

Variable	Likelihood of Having Personally Seen Someone Threatened with a Firearm^a^		Likelihood of Personally Knowing Someone Killed or Injured by a Firearm^b^	
	OR	95% CI	OR	95% CI
Sex				
Male	0.90	0.53–1.50	0.67	0.53–0.84
Female	1.0 (ref)		1.0 (ref)	
Age				
16–18 years	1.12	0.65–1.92	1.19	0.93–1.51
13–15 years	1.0 (ref)		1.0 (ref)	
Residence				
Farm	1.89	0.95–3.77	0.74	0.56–0.96
Country/Not Farm	1.92	0.84–4.35	1.03	0.74–1.44
Town	1.0 (ref)		1.0 (ref)	
Race				
Caucasian	1.0 (ref)		1.0 (ref)	
Non-Caucasian	2.56	1.02–6.40	1.46	0.85–2.49
Rifle/Shotgun in Home				
Yes	0.48	0.21–1.11	1.53	1.06–2.20
No	1.0 (ref)		1.0 (ref)	
Handgun in Home				
Yes	3.01	1.51–6.00	1.23	0.96–1.58
No	1.0 (ref)		1.0 (ref)	

^a^ The total number of cases used in the logistic regression model was 1370

^b^ The total number of cases used in the logistic regression model was 1369

### Know of someone killed or injured by a firearm

Over one-third (36%) of respondents personally knew someone who had been injured or killed by gunfire. See Table 2. Among these respondents, 77% (378/493) knew of at least one person who had died or was injured accidentally (unintentionally) and 30% (148/493) knew of someone who was killed or injured on purpose (e.g. suicide, homicide). Of all study participants, 12% (172/1374) knew of family members, 17% (237/1374) knew of friends and 10% (137/1374) knew of others who had been killed or injured by a firearm. Three had been injured themselves.

A higher percentage of females as compared to males reported personally knowing someone injured or killed by a firearm, *p* = 0.006. Those with a rifle/shotgun (*p* = 0.025) or a handgun (*p* = 0.020) in the home also had higher proportions that personally knew a victim of a firearm injury or death as compared to individuals that did not have firearms in their home.

Logistic regression analysis showed that males and respondents living on farms were 33 and 26% less likely than females and those living in towns, respectively, to know someone who had been injured or killed by gunfire. See Table 3. Those with rifles/shotguns in the home were 1.5 times more likely than those without to have personally known someone killed or injured by a firearm.

### Use of firearms for hunting and shooting sports should be kept legal

Nearly three-quarters of Iowa FFA members in the study strongly agreed (74%) and the vast majority (95%) agreed that the right to use firearms for hunting and shooting sports should be kept legal. See Table 4. Males, older teens, Caucasians, individuals with rifles/shotguns and with handguns in the home, hunters, and those that had fired rifles/shotguns or handguns all had significantly higher proportions that agreed with this statement as compared with their peers. Logistic regression analysis found males were 2.3 times more likely than females, those with rifles/shotguns in their home were 1.9 times more likely than those without, and hunters were 3.2 times more likely than non-hunters to agree that use of firearms for hunting and shooting sports should be kept legal.

**Table 4:** Descriptive, bivariate, and logistic regression analyses of attitudes with respect to the statement, “The right to use firearms for hunting and shooting sports should be kept legal,” among respondents of the Iowa FFA firearm survey

**The right to use firearms for hunting and shooting sports should be kept legal**					
Strongly Agree	1017 (74%)				
Agree	288 (21%)				
Neutral	54 (4%)				
Disagree	12 (1%)				
Strongly Disagree	11 (1%)				
	**Cross Tab Analysis**			**Logistic Regression Analysis**^a^	
Variables	Agree n (Row %)^b^	Neutral/Disagree *n* (Row %)^b^	*p* value	OR	CI
Group N	1305 (94%)	77 (6%)			
Sex			< 0.001		
Male	678 (97%)	19 (3%)		2.33	1.32–4.12
Female	623 (92%)	57 (8%)		1.0 (ref)	
Age			0.041		
16–18 years	861 (95%)	42 (5%)		1.39	0.85–2.25
13–15 years	444 (93%)	35 (7%)		1.0 (ref)	
Residence			0.34		
Farm	690 (95%)	37 (5%)		0.89	0.51–1.57
Country/Not Farm	238 (95%)	12 (5%)		1.26	0.59–2.67
Town	372 (93%)	28 (7%)		1.0 (ref)	
Race			0.009		
Caucasian	1251 (95%)	69 (5%)		1.0 (ref)	
Non-Caucasian	53 (87%)	8 (13%)		0.45	0.19–1.10
Rifle/Shotgun in Home			< 0.001		
Yes	1111 (96%)	48 (4%)		1.88	1.01–3.52
No	194 (87%)	29 (13%)		1.0 (ref)	
Handgun in Home			0.001		
Yes	768 (96%)	34 (4%)		0.99	0.56–1.74
No	537 (93%)	43 (7%)		1.0 (ref)	
Hunted			< 0.001		
Yes	741 (98%)	16 (2%)		3.18	1.72–5.89
No	552 (90%)	60 (10%)		1.0 (ref)	
Fired Rifle/Shotgun			< 0.001	Not used in the analysis	
> 20 times	782 (97%)	21 (3%)			
6–20 times	158 (97%)	5 (3%)			
1–5 times	191 (92%)	16 (8%)			
Never	171 (84%)	33 (16%)			
Fired a Handgun			< 0.001	Not used in the analysis	
> 20 times	408 (98%)	7 (2%)			
6–20 times	171 (98%)	4 (2%)			
1–5 times	242 (95%)	14 (5%)			
Never	474 (91%)	46 (9%)			

^a^ The total number of cases used in the logistic regression model was 1358

^b^ The sum of n for a variable may not equal the total Group N due to missing values

### Safety course should be required for a hunting license

Over 60% strongly agreed and 89% overall agreed that a firearm safety course should be required to get a hunting license. See Table 5. Males, those with a handgun in the home, and those that had fired a rifle/shotgun > 20 times all had significantly greater percentages that did not agree with this statement. Males were half as likely as females to agree that a safety course should be mandatory to get a hunting license.

**Table 5:** Descriptive, bivariate, and logistic regression analyses of attitudes with respect to the statement, “A firearm safety course should be required to get a hunting license” among respondents of the Iowa FFA firearm survey

**A firearm safety course should be required to get a hunting license**					
Strongly Agree	861 (62%)				
Agree	375 (27%)				
Neutral	105 (8%)				
Disagree	31 (2%)				
Strongly Disagree	10 (1%)				
	**Cross Tab Analysis**			**Logistic Regression Analysis**^a^	
Variables	Agree *n* (Row %)^b^	Neutral/Disagree *n* (Row %)^b^	*p* value	OR	CI
Group N	1236 (89%)	146 (11%)			
Sex			< 0.001		
Male	604 (87%)	93 (13%)		0.50	0.34–0.73
Female	629 (93%)	51 (8%)		1.0 (ref)	
Age			0.30		
16–18 years	802 (89%)	101 (11%)		0.92	0.63–1.35
13–15 years	434 (91%)	45 (9%)		1.0 (ref)	
Residence			0.73		
Farm	646 (89%)	81 (11%)		0.88	0.58–1.35
Country/Not Farm	225 (90%)	25 (10%)		1.03	0.59–1.78
Town	361 (90%)	39 (10%)		1.0 (ref)	
Race			0.81		
Caucasian	1181 (89%)	139 (11%)		1.0(ref)	
Non-Caucasian	54 (89%)	7 (11%)		0.99	0.41–2.39
Rifle/Shotgun in Home			0.12		
Yes	1030 (88%)	128 (12%)		0.83	0.44–1.55
No	206 (92%)	17 (8%)		1.0 (ref)	
Handgun in Home			0.046		
Yes	706 (97%)	96 (3%)		0.72	0.48–1.08
No	530 (91%)	50 (9%)		1.0 (ref)	
Hunted			0.55		
Yes	674 (89%)	83 (11%)		1.27	0.85–1.90
No	551 (90%)	61 (10%)		1.0 (ref)	
Fired Rifle/Shotgun			0.043	Not used in the analysis	
> 20 times	705 (88%)	98 (12%)			
6–20 times	153 (94%)	10 (6%)			
1–5 times	187 (90%)	20 (10%)			
Never	189 (93%)	15 (7%)			
Fired a Handgun			0.61	Not used in the analysis	
> 20 times	366 (88%)	49 (12%)			
6–20 times	160 (91%)	15 (9%)			
1–5 times	231 (90%)	25 (10%)			
Never	469 (90%)	51 (10%)			

^a^ The total number of cases used in the logistic regression model was 1358

^b^ The sum of n for a variable may not equal the total Group N due to missing values

### Background check should be required to buy a firearm

Ninety percent agreed with 60% strongly agreeing that there should be a background check required by law before someone can buy a firearm. See Table 6. Hunters and those who had fired a handgun > 5 times had significantly lower proportions that agreed with the statement. There were no significant differences in logistic regression results.

**Table 6:** Descriptive, bivariate, and logistic regression analyses of attitudes with respect to the statement, “There should be a background check required by law before someone can buy a firearm” among respondents of the Iowa FFA firearm survey

**There should be a background check required by law before someone can buy a firearm**					
Strongly Agree	823 (60%)				
Agree	408 (30%)				
Neutral	121 (9%)				
Disagree	18 (1%)				
Strongly Disagree	11 (1%)				
	**Cross Tab Analysis**			**Logistic Regression Analysis**^a^	
Variables	Agree *n* (Row %)^b^	Neutral/Disagree *n* (Row %)^b^	*p* value	OR	CI
Group N	1231 (89%)	150 (11%)			
Sex			0.098		
Male	612 (88%)	85 (12%)		0.84	0.58–1.20
Female	615 (91%)	64 (9%)		1.0 (ref)	
Age			0.73		
16–18 years	803 (89%)	100 (11%)		1.00	0.69–1.44
13–15 years	428 (90%)	50 (10%)		1.0 (ref)	
Residence			0.54		
Farm	642 (88%)	85 (12%)		0.77	0.50–1.17
Country/Not Farm	223 (89%)	27 (11%)		0.87	0.51–1.49
Town	361 (90%)	38 (10%)		1.0(ref)	
Race			0.16		
Caucasian	1224 (90%)	133 (10%)		1.0 (ref)	
Non-Caucasian	51 (84%)	10 (16%)		0.60	0.28–1.26
Rifle/Shotgun in Home			0.68		
Yes	1030 (89%)	128 (11%)		1.00	0.56–1.80
No	201 (90%)	22 (10%)		1.0 (ref)	
Handgun in Home			0.92		
Yes	713 (89%)	88 (11%)		1.09	0.74–1.61
No	518 (89%)	62 (11%)		1.0 (ref)	
Hunted			0.034		
Yes	663 (88%)	94 (12%)		0.70	0.47–1.05
No	557 (91%)	54 (9%)		1.0 (ref)	
Fired Rifle/Shotgun			0.19	Not used in the analysis	
> 20 times	704 (88%)	98 (12%)			
6–20 times	149 (91%)	14 (9%)			
1–5 times	189 (91%)	18 (9%)			
Never	187 (92%)	17 (8%)			
Fired a Handgun			0.018	Not used in the analysis	
> 20 times	363 (87%)	52 (13%)			
6–20 times	152 (87%)	23 (13%)			
1–5 times	242 (95%)	14 (15%)			
Never	465 (90%)	54 (10%)			

^a^ The total number of cases used in the logistic regression model was 1357

^b^ The sum of n for a variable may not equal the total Group N due to missing values

### Safe firearm storage should be required by law

Over 60% agreed, with one-third strongly agreeing, that there should be laws that require safe storage (locked and unloaded) of firearms in the home. See Table 7. Those with significantly lower proportions agreeing with this statement as compared to their peers included males, older teens, those living on farms and in the country but not on a farm, individuals who reported unsafe storage of rifles/shotguns or of handguns in their home, hunters, and those who had fired rifles/shotguns or handguns > 5 times. The lowest percentage (46%) agreeing with safe storage requirements included those who reported unsafe storage of rifles/shotguns or of handguns at least some of the time in their home. In logistic regression analysis, males were 50% less likely than females, those from farms were 30% less likely as those in town, and hunters were 40% less likely than non-hunters to agree that safe firearm storage in the home should be required by law. In addition, adolescents reporting unsafe storage of rifles/shotguns and of handguns in their home were 70 and 30% less likely, respectively, to agree safe firearm storage should be required by law as compared to individuals reporting safe storage.

**Table 7:** Descriptive, bivariate, and logistic regression analyses of attitudes with respect to the statement, “There should be laws that require safe storage (locked and unloaded) of firearms in homes,” among respondents of the Iowa FFA firearm survey

**There should laws that require safe storage (locked and unloaded) of firearms in homes**					
Strongly Agree	453 (33%)				
Agree	396 (29%)				
Neutral	347 (25%)				
Disagree	114 (8%)				
Strongly Disagree	72 (5%)				
	**Cross Tab Analysis**			**Logistic Regression Analysis**^a^	
Variables	Agree *n* (Row %)^b^	Neutral/Disagree *n* (Row %)^b^	*p* value	OR	CI
Group N	841 (61%)	533 (39%)			
Sex			< 0.001		
Male	354 (51%)	343 (49%)		0.53	0.41–0.69
Female	494 (73%)	186 (27%)		1.0 (ref)	
Age			0.006		
16–18 years	531 (59%)	372 (41%)		0.84	0.65–1.10
13–15 years	318 (66%)	161 (34%)		1.0 (ref)	
Residence			< 0.001		
Farm	411 (57%)	316 (43%)		0.70	0.52–0.93
Country/Not Farm	154 (62%)	96 (38%)		0.83	0.57–1.21
Town	281 (70%)	119 (30%)		1.0 (ref)	
Race			0.90		
Caucasian	811 (61%)	509 (39%)		1.0(ref)	
Non-Caucasian	37 (61%)	24 (39%)		0.60	0.32–1.11
Rifle/Shotgun Storage			< 0.001		
No Rifles/Shotguns	186 (83%)	37 (17%)		1.04	0.63–1.69
Unsafe Storage	327 (46%)	389 (54%)		0.33	0.24–0.45
Safe Storage	270 (75%)	90 (25%)		1.0 (ref)	
Unsure Storage	65 (79%)	17 (21%)		1.06	0.53–2.13
Handgun in Home			< 0.001		
No Handguns	406 (70%)	174 (30%)		0.99	0.69–1.42
Unsafe Storage	211 (46%)	252 (54%)		0.67	0.47–0.96
Safe Storage	188 (68%)	87 (32%)		1.0 (ref)	
Unsure Storage	43 (68%)	20 (32%)		0.61	0.30–1.24
Hunted			< 0.001		
Yes	391 (52%)	366 (48%)		0.61	0.46–0.80
No	449 (73%)	163 (27%)		1.0 (ref)	
Fired Rifle/Shotgun			< 0.001	Not used in the analysis	
> 20 times	404 (50%)	399 (50%)			
6–20 times	118 (72%)	45 (28%)			
1–5 times	161 (78%)	46 (22%)			
Never	165 (81%)	39 (19%)			
Fired a Handgun			< 0.001	Not used in the analysis	
> 20 times	195 (47%)	220 (53%)			
6–20 times	97 (55%)	78 (45%)			
1–5 times	169 (66%)	87 (34%)			
Never	380 (73%)	140 (27%)			

^a^ The total number of cases used in the logistic regression model was 1358

^b^ The sum of *n* for a variable may not equal the total Group N due to missing values

## Discussion

We found that about one in 20 Iowa FFA member respondents had seen someone threatened or personally been threatened with a firearm, and over one-third personally knew someone who was killed or injured by gunfire. Gun violence, especially mass shootings in schools and teen suicides, have brought the issue of gun control to the forefront for many adolescents and teens yet their perspective has rarely been gathered. In one study, over 90% of high school students reported having been exposed to gun policy issues through the media and four-fifths had discussed gun control within the last year in class, at home and/or with friends (Vittes et al., 2003). The majority of rural youth in our study supported each of the firearm safety policies presented.

### Negative personal experiences with firearms

Many U.S. adolescents have negative personal experiences with firearms. Studies in the past have often concentrated on urban youth because of the high incidence of homicides in the inner city (Srinivasan et al., 2014; Teplin et al., 2014). One survey of 10 inner city high schools in four states found that one-fifth had been threatened with a gun and 12% had been shot at (Sheley et al., 1992), and another study of three public New York City high schools with a majority of Hispanic students showed nearly a quarter had been threatened and almost half having seen someone threatened by a firearm. A national sample of high school students found that one-third knew someone who had been threatened with a firearm or shot at in school (Vittes et al., 2003). The 5% of Iowa FFA members who personally witnessed someone being threatened with a firearm was significantly lower than these studies but demonstrates that the negative impact of firearm violence is not limited to urban areas.

FFA members of non-Caucasian races in our study were over twice as likely to report having personally seen someone threatened with a firearm as compared to Caucasian members. A previous study of a rural North Carolina county found African Americans to more commonly be victims of fatal and non-fatal firearm injury than those that were White (Sadowski & Munoz, 1996). The number of adolescents of non-Caucasian races in our study was limited and further study is needed to evaluate firearm violence by race in rural areas.

Our study also showed that those with a handgun in their home (58% of respondents) were three times more likely than those without to have personally seen someone threatened with a firearm. Handguns are often reported to be kept for protection (Siegel & Boine, 2020), and thus are frequently stored unlocked and/or loaded for quick access (Aitken et al., 2020). Unfortunately, handguns in the home are much more likely to kill or injure a household member than be used in self-defense (Anglemyer et al., 2014; Kellermann & Reay, 1986; Kellermann et al., 1993). Additionally, studies have found that the difference between interpersonal violence rates between rural and urban areas are not as large as some assume or in some cases, no different (Murphy, 2018; Osgood & Chambers, 2003).

Almost 40% of respondents personally knew someone who had been injured or killed by gunfire. Of those, almost one-third and over three-quarters knew of someone killed or injured intentionally or unintentionally, respectively. In a national survey, one-quarter of high school students reported that someone had been killed or seriously injured by gunfire in their neighborhood (Vittes et al., 2003). Both firearm-related unintentional injuries and suicides are more frequent in rural than in urban areas (Herrin et al., 2018; Branas et al., 2004; Nance et al., 2010; Carr et al., 2012; Fontanella et al., 2015; Nestadt et al., 2017; Kegler et al., 2017; Ivey-Stephenson et al., 2017). Major factors in this may be the greater prevalence of firearms in rural homes (Parker et al., 2017; Nordstrom et al., 2001), and the fact that hunting is significantly more prevalent among rural than urban residents (U.S. Department of the Interior, U.S, 2016).

### Attitudes toward firearm safety policies

Although nationally most high school students support more restrictive firearm policies, the majority do not support total banning of firearm ownership, including handguns (Vittes et al., 2003; Kahn et al., 2001; Van Sparrentak et al., 2018). We similarly found that three-quarters of adolescents in our study strongly agreed that the right to use firearms for hunting and shooting sports should be kept legal. This support was stronger among males, those with rifles/shotguns used for hunting in their homes, and those who hunted themselves.

Many consider firearm safety education a critical component of firearm injury prevention. In many states, including Iowa, completion of a hunter safety course is a state requirement to obtain a hunting license (American Hunting Lease Association, 2020). However, training requirements vary significantly from state to state, particularly related to minors accompanied by adults. As a significant proportion of unintentional firearm-related injuries in rural areas are due to hunting incidents (Dodge et al., 1994; Guetschow et al., 2018; Carter, 1989), mandatory safety training seems justified. Nearly 90% of our survey respondents agreed.

Background check laws are designed to reduce gun violence by preventing purchase of firearms by those considered high risk of being a danger to themselves or others. Although the Brady Handgun Violence Prevention Act imposed federal requirements for background checks on sales by licensed firearm dealers, it did not cover private sales and transfers. Only some states have universal background checks that expand the Brady Act to include these sales/transfers. A 2015 national survey of gun owners found that only 20% of purchases in the previous two years included undergoing a background check (Miller et al., 2017). Again, nearly all (90%) of the rural teenagers in our study agreed that laws should require background checks for all firearm purchases and only 2% disagreed. Other studies of adults and youth have also found nearly unanimous support for universal background checks (Poll, 2019; Vittes et al., 2003; Smith, 2002; Sorensen, 2015). Youth in a national survey were especially concerned about those with mental illness, a criminal record, or a history of violence getting access to firearms (Sorensen, 2015).

About one-third of U.S. homes with children and teenagers have a firearm (Azrael et al., 2018; Schuster et al., 2000; Johnson et al., 2004), but this is even higher in most rural communities (Sadowski & Munoz, 1996; Parker et al., 2017; Nordstrom et al., 2001; Senturia et al., 1994; Shaughnessy et al., 1999). Frequently, these firearms are stored unlocked and/or loaded (Azrael et al., 2018; Hamilton et al., 2018a). The vast majority of unintentional firearm injuries in children occur in the home (Faulkenberry & Schaechter, 2015), and having access to a firearm markedly increases suicide risk for adults and teens (Anglemyer et al., 2014; Dahlberg et al., 2004; Miller et al., 2006; Miller et al., 2015; Kung et al., 2005; Shenassa et al., 2004; Grossman et al., 2005; Hemenway, 2011; Stroebe, 2013). Secure firearm storage has been associated with a reduction in unintentional firearm deaths in children (Miller et al., 2005). Moreover, child access prevention (CAP) laws pertaining to proper storage of firearms in a home have been found to significantly reduce firearm fatality rates in children younger than 15 years old (Azad et al., 2020).

Two-thirds of firearm owners and 90% of non-owners state that all firearms should be kept locked when there are children in the home (Parker et al., 2017). Nearly three-quarters in national studies of adults and of youth supported CAP laws requiring safe gun storage with adult support increasing over time (Vittes et al., 2003; Barry et al., 2019). High schoolers wanted adults to be criminally liable when their storage practices allowed firearm access to a child, which is currently a law in a minority of states (Vittes et al., 2003). In Iowa, individuals are only criminally liable if a minor lawfully gains access to a firearm without consent and exhibits it in a public place in an unlawful manner or uses the firearm unlawfully to cause injury or death to any person. In our study, the majority of adolescents (62%) agreed that there should be laws requiring safe storage and only 13% disagreed.

As in other surveys (Smith, 2002), females in our study were more likely than males to support laws requiring safe firearm storage. This gender gap is commonly seen in studies related to stronger gun policies (Vittes et al., 2003; Parker et al., 2017; Smith, 2002). We also noted our respondents living on farms were less supportive of these laws than adolescents living in towns, as were participants reporting unsafe storage of firearms in their home as compared to those with firearms stored safely. A previous study similarly found residents of rural areas to be less favorable of safe firearm storage requirements (Smith, 2002).

The American Academy of Pediatrics (AAP) has highlighted the importance of ensuring the safe storage of firearms in homes (Dowd & Sege, 2012). A key component in achieving this and protecting children equally across states would be the passage and enforcement of universal CAP laws (Azad et al., 2020; Hamilton et al., 2018b; Santaella-Tenorio et al., 2016; Cummings et al., 1997; Webster et al., 2004; Webster & Starnes, 2000; Lee et al., 2013; Hepburn et al., 2006; DeSimone et al., 2013). Surveys of members of the National Association of Social Workers and the AAP Council on Child Abuse and Neglect showed strong support for CAP law passage (Evans et al., 2017; Jennissen et al., 2018). Strict enforcement of laws holding gun-owners accountable when children and adolescents access firearms is a critical step in reducing firearm access by youth and the firearm-related deaths and injuries that subsequently occur.

### Limitations

Our study is limited in that it was performed in a single Midwest state with a primarily Caucasian population, and most subjects were from rural communities. Therefore, our results may not be generalizable to other areas of the country, especially urban and suburban localities. Our findings may also not be representative of all rural regions of the state as a convenience sample of adolescents attending a state FFA conference was used rather than a randomized sample. However, the vast majority of Iowa’s counties were represented by subjects in the study. Although we observed some differences when we compared Caucasians to non-Caucasians, the numbers of the latter were very small and represented multiple races, so caution should be used in interpreting these data. Similar to other surveys, our data may be subject to bias because of inaccurate recall, lack of knowledge, or social desirability bias. Data were collected anonymously which should have decreased the latter.

## Conclusions

The majority of youth in this study supported firearm safety measures including required training, background checks, and safe firearm storage in homes. These findings are consistent with the national youth-led call for firearm safety. However, certain demographic factors are associated with differing attitudes regarding firearms and may be important to address in firearm injury prevention efforts. Additionally, over one-third of rural adolescent respondents personally knew someone who was killed or injured by a firearm and 5% had seen someone or been personally threatened with firearm violence. Although our study did not investigate the effects of this firearm violence on participants’ mental health and wellbeing, future studies addressing this question seem highly justified.

## Data Availability

Data and materials are available to other parties for research purposes after a data sharing agreement plan is agreed to and signed. Those interested should contact the corresponding author.

## Abbreviations

AAP: American Academy of Pediatrics
CAP: Child access prevention
e.g.: Exempli gratia (for example)
FFA: Formerly stood for Future Farmers of America
TM: Trademark
U.S.: United States
